# Self-Dissolving Microneedle Arrays for Transdermal Absorption Enhancement of Human Parathyroid Hormone (1-34)

**DOI:** 10.3390/pharmaceutics10040215

**Published:** 2018-11-04

**Authors:** Chihiro Naito, Hidemasa Katsumi, Tomoko Suzuki, Ying-shu Quan, Fumio Kamiyama, Toshiyasu Sakane, Akira Yamamoto

**Affiliations:** 1 Department of Biopharmaceutics, Kyoto Pharmaceutical University, Yamashina-ku, Kyoto 607-8414, Japan; kd16008@poppy.kyoto-phu.ac.jp (C.N.); suzuki_t1130@yahoo.co.jp (Tom.S.); sakane@kobepharma-u.ac.jp (Tos.S.); yamamoto@mb.kyoto-phu.ac.jp (A.Y.); 2 CosMED Pharmaceutical Co., Ltd., Minami-ku, Kyoto 601-8014, Japan; quan@cosmed-pharm.co.jp (Y.-s.Q.); kamiyama@cosmed-pharm.co.jp (F.K.); 3 Department of Pharmaceutical Technology, Kobe Pharmaceutical University, Higashinada-ku, Kobe 658-8558, Japan

**Keywords:** transdermal absorption enhancement, microneedle arrays, human parathyroid hormone 1-34, bioavailability

## Abstract

Human parathyroid hormone (1-34) (PTH) has been widely used as the subcutaneous injection formulation for the treatment of osteoporosis. In the present study, we developed an efficient transdermal delivery system of PTH by using dissolving microneedle arrays (MNs) composed of hyaluronic acid (HA) for the treatment of osteoporosis. PTH-loaded MNs, with needle length 800 µm, were fabricated via a micro-molding method. The stability of PTH in MNs was found to be 6-fold higher than that of PTH solution when stored at room temperature (15–20 °C) for one month. Micron-scale pores were clearly visible in rat skin following application of PTH-loaded MNs. PTH-loaded MNs were completely dissolved by 60 min following application to rat skin. The bioavailability (BA) of PTH relative to subcutaneous injection was 100 ± 4% following application of PTH-loaded MNs in rats. In addition, PTH-loaded MNs were found to effectively suppress decreases in bone density in a rat model of osteoporosis. Furthermore, no skin irritation was observed at the site of application in rats. These findings indicate that our dissolving MNs have a potential use in formulations for the transdermal delivery of PTH and for the treatment of osteoporosis.

## 1. Introduction

Human recombinant parathyroid hormone (1-34) (PTH) is a peptide that consists of 34 amino acids. It is widely used for the treatment of osteoporosis. PTH is unique in that intermittent administration of this peptide leads to an increase in bone formation and bone mass. This is a result of its anabolic effect, which is not a characteristic of other osteoporosis therapeutics that are currently available [1]. In contrast, because of the low absorption characteristics of peptides and proteins such as PTH from the gastrointestinal tract [2], the route of delivery of PTH has been limited to a once-daily or once-weekly subcutaneous injection [3,4]. However, frequent injections are both inconvenient and painful for patients and reduce quality of life (QOL). Therefore, an alternative approach for PTH administration is clearly needed.

Transdermal drug delivery has several advantages. It is painless and allows ease of application. Therefore, transdermal delivery is considered to be an attractive method for the delivery of PTH. However, the stratum corneum, the outermost layer of the skin, restricts the permeation of hydrophilic and macromolecular drugs [5,6]. In order to overcome this barrier function of the stratum corneum, several approaches including the use of chemical enhancers, iontophoresis, electroporation, sonophoresis, jet injection, and microneedle arrays (MNs) have been trialed [7,8,9,10,11].

Of the various strategies available, MNs are considered to be an efficient approach for the transdermal delivery of PTH. MNs are composed of micron-scale needles arrayed onto a transdermal patch. MNs enhance the permeation of macromolecules and hydrophilic compounds by creating micron-sized pores in the skin [12,13,14,15]. MNs also avoid the risk of infection as they are only minimally invasive compared to needle injections that necessarily puncture through to deeper layers of the skin. Furthermore, MNs are easy to administer, like transdermal patches. [16]

ZP-PTH^®^, a PTH-coated titanium microneedle patch system, with needle length 190 µm, was developed for the treatment of osteoporosis [3]. However, the efficient transdermal delivery of PTH is still difficult owing to short needles. In addition, it has previously been reported that the needles may be accidentally broken and remain in the skin following the application of solid-type MNs fabricated from metal, silicon, or glass [17,18,19]. Therefore, the development of a new type of MNs is needed for the efficient and safe transdermal delivery of PTH. Recently, we successfully developed a transdermal delivery system for insulin by using dissolving MNs, with needle length 800 µm, composed of hyaluronic acid (HA) [20]. Furthermore, broken needles remaining in the skin were not found to be a concern with our MNs, as they dissolved completely in the skin following application. As a result of these findings, we suggest that our dissolving MNs composed of HA may have potential as transdermal formulations for efficient and safe PTH delivery for the treatment of osteoporosis. However, there are large differences between the structure, stability, pharmacokinetics, and pharmacological activities of insulin and PTH, and transdermal delivery of PTH using dissolving MNs has not previously been investigated.

In the present study, we designed and developed dissolving MNs composed of HA as the base material for the efficient transdermal delivery of PTH. The stability of PTH in the MNs and the process by which MNs dissolve in the skin were evaluated. Pharmacokinetic analyses were performed in order to evaluate the transdermal absorption of PTH following application of PTH-loaded MNs. In addition, the therapeutic potential of MNs to treat post-menopausal osteoporosis following the application of PTH-loaded MNs was assessed in a rat osteoporosis model. The risk of skin irritation following application of PTH-loaded MNs was also assessed.

## 2. Materials and Methods

### 2.1. Materials

HA was purchased from Kikkoman Biochemifa Company (Tokyo, Japan). PTH was supplied from CosMED Pharmaceutical Co., Ltd. (Kyoto, Japan). The SEP-Column containing 200 mg C18 was purchased from Phoenix Pharmaceuticals, Inc. (Burlingame, CA, USA). Trypan blue was purchased from Nacalai Tesque, Inc. (Kyoto, Japan). Tissue-TeK^®^ O.C.T. Compound was purchased from Sakura Finetek Japan Co., Ltd. (Tokyo, Japan). Paraformaldehyde, ethylenediaminetetraacetic acid (EDTA), and aprotinin were purchased from Wako Pure Chemical Industries, Ltd. (Tokyo, Japan). All other chemicals were obtained commercially as reagent-grade products.

### 2.2. Animals

Male Wistar rats (250–270 g) and female Sprague-Dawley rats (180–200 g) were purchased from Japan SLC, Inc. (Shizuoka, Japan). All animal experiments were conducted in accordance with the principles and procedures outlined in the National Institutes of Health Guidelines for the Care and Use of Laboratory Animals. The protocols for animal experiments were approved by the Animal Experimentation Committee of the Kyoto Pharmaceutical University.

### 2.3. Fabrication of PTH-Loaded MNs

The MNs were fabricated by using micromolding technologies with HA as a base material [20,21], and then, were loaded with PTH. In brief, a 15% HA solution was obtained by mixing well in distilled water. The PTH solution was added to the 15% HA solution under acidic conditions and mixed well to prepare a HA solution containing PTH. Of the resulting HA solution containing PTH, 0.3 mL was placed on a 2 cm × 2 cm micromold at room temperature. After 2 h drying in a desiccator, the remaining solution was removed from the surface of mold with cotton, then 0.1 mL of 20% HA solution was placed on the same place as the micromold. After drying the micromold completely, a 2 cm × 2 cm polyethylene terephthalate (PET) adhesive tape was attached on the baseplate for reinforcing. A sheet of PTH-loaded MNs was obtained by peeling the mold off. PTH-loaded MNs in circular area with a diameter of 10 mm were obtained by cutting the sheet with a punch.

### 2.4. Determination of Drug Contents in PTH-Loaded MNs

In order to confirm the PTH content in the needle part of MNs, the needles were separated from the back layers of MNs and dissolved in 5 mL of phosphate buffered saline (PBS, pH 7.4) in a vial. The solution was then stirred for 1 h at 72 rpm at 32 °C [22]. Concentrations of PTH in the samples were measured using the PTH (1-34) ELISA kit (PTH (1-34), Human, EIA Kit, High Sensitivity, Peninsula Laboratories International, Inc. San Carlos, CA, USA)

### 2.5. Stability of PTH in MNs Fabricated from HA

PTH-loaded MNs were packaged into an aluminum sachet purged with nitrogen and stored at −20 °C, 4 °C, or at room temperature (15–20 °C) [23]. PTH solution dissolved in PBS was kept at the same temperatures as the control. Following one month of storage, the needles were separated from the back layers of MNs and dissolved in PBS. The actual amounts of PTH present in the needle parts of the MNs were measured using the PTH (1-34) ELISA kit (PTH (1-34), Human, EIA Kit, High Sensitivity, Peninsula Laboratories International, Inc. San Carlos, CA, USA).

### 2.6. Skin Piercing Ability of MNs

The abdominal skin of Wistar rats was shaved while the animals were placed under pentobarbital anesthesia (52 mg/kg as pentobarbital sodium salt). PTH-loaded MNs were then applied to the abdominal skin of rats. Following a 1 h application, the MNs were removed and the application sites were stained with 0.4% trypan blue in water for 10 min. Following the removal of the trypan blue solution with tissue paper, the surface of the skin with applied MNs was observed. The areas of skin to which MNs had been applied were then excised from euthanized rats and embedded in optimal cutting temperature (OCT) compound. The embedded skin samples were frozen in liquid nitrogen and sectioned into 10 µm slices using a cryomicrotome (CM1950; Leica, Wetzlar, Germany). The sections were then visualized under a microscope (BIOZERO^®^; KEYENCE, Osaka, Japan) [21,24].

### 2.7. Effect of PTH-Loaded MNs on Transepidermal Water Loss (TEWL)

The abdominal skin of Wistar rats was shaved while the animals were placed under pentobarbital anesthesia (52 mg/kg as pentobarbital sodium salt). PTH-loaded MNs were then applied to the abdominal skin of rats. Following a 1 h application, the MNs were removed. The TEWL values of rat skin were measured using a Tewameter (TM 300, Courage and Khazaka Electronic GmbH, Cologne, Germany). This was done by applying a probe to the skin until a stable reading was obtained. TEWL values were determined before and directly following the removal of MNs at indicated time intervals over a period of 48 h. Separately, a group of rats were treated by tape stripping 20 times each as a positive control. Untreated rats were used as a negative control. TEWL values were also determined over similar time intervals for both the positive and negative control groups [25].

### 2.8. Dissolution of MNs Following Application in Rats

The abdominal skin of Wistar rats was shaved while the animals were placed under pentobarbital anesthesia (52 mg/kg as pentobarbital sodium salt). PTH-loaded MNs were then applied to the abdomen of rats. At pre-determined times, the MNs were removed and visualized using a digital microscope (Shodensha, Osaka, Japan) [24].

### 2.9. In Vivo Transdermal Delivery of PTH

The abdominal skin of Wistar rats was shaved while the animals were placed under pentobarbital anesthesia (52 mg/kg as pentobarbital sodium salt). PTH-loaded MNs (20 µg/rat) were then applied to the abdominal skin of rats. The dosage of PTH in MNs was calculated based on the content of PTH in the needle part of MNs. Separately, PTH solution (20 µg/rat) was subcutaneously injected into the backs of rats. At pre-determined intervals, 0.3 mL of blood was withdrawn from the jugular veins while the animals were placed under pentobarbital anesthesia (52 mg/kg as pentobarbital sodium salt) and collected in tubes containing 0.3 mg of EDTA and 150 KIU of aprotinin. The collected blood was then centrifuged for 15 min at 4000 rpm to obtain plasma. The plasma samples were then stored at −80 °C until required for determination of PTH concentration. The peak PTH plasma concentration (C_max_) and time to reach C_max_ (T_max_) were determined directly from the plasma concentration–time profiles. Area under the plasma concentration-time curve (AUC) after transdermal administration or subcutaneous injection was calculated by the trapezoidal formula and extrapolation to infinite time based on monoexponential equation [26].

### 2.10. The Determination of PTH Concentrations in Plasma

The PTH in the plasma sample was extracted and purified using SEP-columns. The solution obtained was lyophilized and dissolved in EIA buffer. Concentrations of PTH were determined using the PTH (1-34) ELISA kit (PTH (1-34), Human, EIA Kit, High Sensitivity, Peninsula Laboratories International, Inc. San Carlos, CA, USA).

### 2.11. Skin Irritation Following Application of MNs in Rats

PTH-loaded MNs were applied to the abdomen of rats (20 µg/rat). The MNs were then removed 1 h after their application. Skin irritation was assessed by observation of the application site of rats at pre-determined times [21,24].

### 2.12. Therapeutic Potential of PTH-Loaded MNs for Treatment of Osteoporosis

The post-menopausal rat model of osteoporosis was established by performing ovariectomies (OVX) of female Sprague-Dawley rats [21]. Four weeks following OVX, PTH-loaded MNs (20 µg/rat) were applied to the rats twice a week, for a period of 4 weeks. Separately, PTH (20 µg/rat) was subcutaneously injected into the rats. Eight weeks after OVX, rats were euthanized, and the tibias were excised and fixed in 4% paraformaldehyde in PBS. The fixed tibias were then embedded in paraffin and sectioned by microtome. The sections of tibias were subsequently stained with hematoxylin and eosin and then used for histological analysis.

## 3. Results

### 3.1. Fabrication of PTH-Loaded MNs

**Figure 1:**
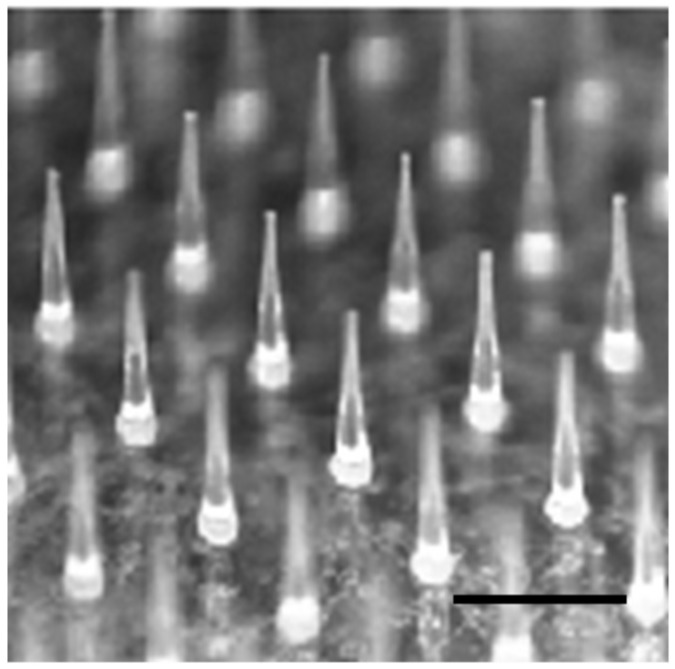
Typical photomicrograph of human parathyroid hormone (1-34) (PTH)-loaded microneedle arrays (MNs). Scale bar represents 800 µm.

Figure 1 shows a typical photomicrograph of PTH-loaded MNs. Each needle was approximately 800 µm in height, with a diameter of 160 µm at the base and 40 µm at the tip, and there was an interspacing of 600 µm between each row of needles. The MNs had approximately 220 needles positioned within a circular area with a diameter of 10 mm. The content of PTH in the needle part of MNs was 20.5 ± 3.0 µg.

### 3.2. Stability of PTH in MNs Fabricated from HA

**Figure 2:**
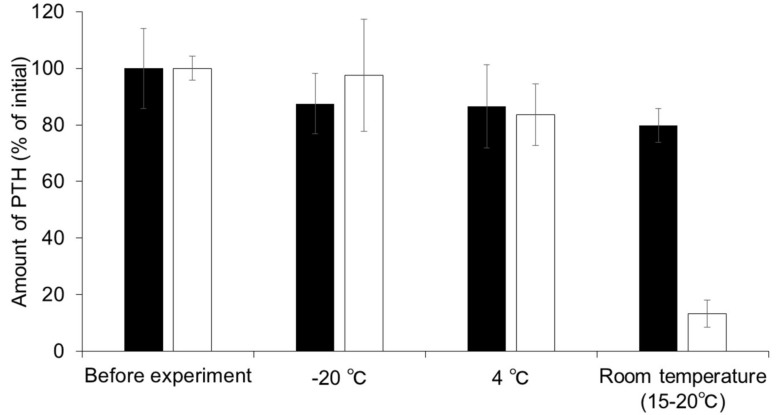
Stability of human parathyroid hormone (1-34) (PTH) in microneedle arrays (MNs) and PTH solution stored at −20, 4, and 15–20 °C for one month. Keys: (■) PTH-loaded MNs and (□) PTH solution. Results are expressed as the means ± standard deviation (SD) of three experiments.

Figure 2 shows the stability of PTH in MNs for one month at either −20 °C, 4 °C, or at room temperature (15–20 °C). The concentration of PTH in PBS was found to decrease over time and approximately 98%, 84%, or 13% of the initial PTH was found to be retained in the solution after storage for one month at −20 °C, 4 °C, or room temperature, respectively. Although no significant difference was observed between the amount of PTH in MNs compared to PTH in solution at −20 °C and 4 °C, PTH in MNs was found to be retained at a level 6-fold higher than that in solution following storage for one month at room temperature.

### 3.3. Skin Piercing Ability of PTH-Loaded MNs

**Figure 3:**
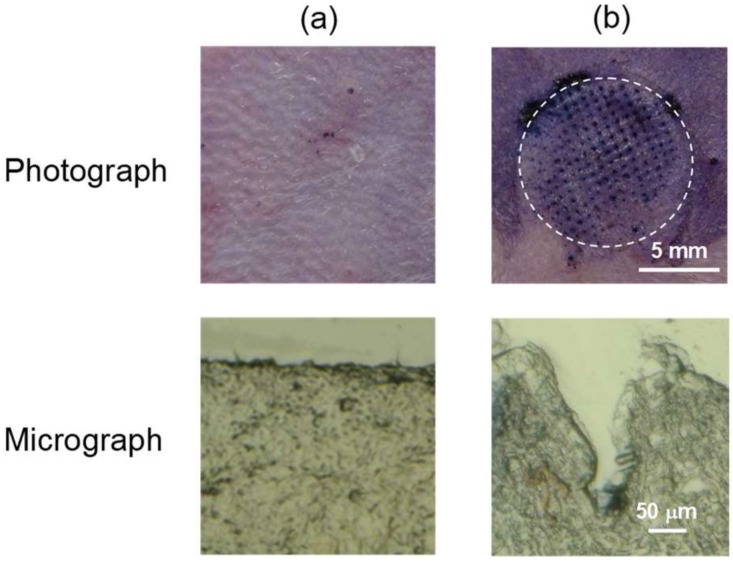
Photograph and micrograph of rat skin following application of human parathyroid hormone (1-34) (PTH)-loaded microneedle arrays (MNs). (**a**) Untreated and (**b**) PTH-loaded MNs.

Figure 3 shows the surface images of rat skin stained with trypan blue and frozen section images following the application of PTH-loaded MNs. A large number of micron-scale pores (150 µm in depth) were clearly observed on the rats’ skin following the application of MNs.

### 3.4. Evaluation of Skin Barrier Function Following Application of PTH-Loaded MNs

**Figure 4:**
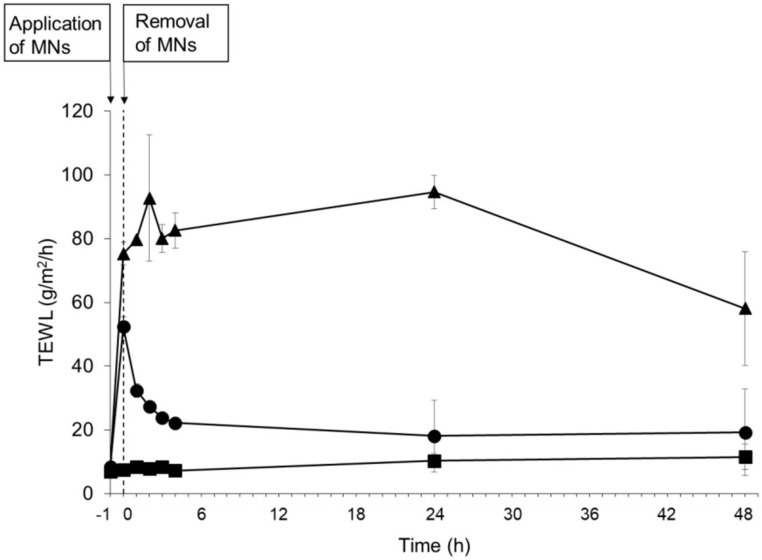
Effects of human parathyroid hormone (1-34) (PTH)-loaded microneedle arrays (MNs) application on transepidermal water loss (TEWL) of rat skin in vivo. Keys: (●) PTH-loaded MNs, (▲) tape stripping treatment, and (■) intact skin. Results are expressed as the means ± standard deviation (SD) of three rats.

To evaluate the piercing ability of MNs and the recovery of the skin barrier following the application of MNs to the skin, the TEWL values of rat skin were measured as an indicator of the barrier function of the stratum corneum. Figure 4 shows the TEWL value before and after application of MNs. The TEWL value of intact skin was 8.42 ± 2.76 g/m^2^/h. The TEWL significantly increased and reached a level of 75.30 ± 3.61 g/m^2^/h immediately following the tape stripping as a positive control. Although the mean TEWL value transiently increased and reached a level of 52.30 ± 3.16 g/m^2^/h immediately following the 1 h application of MNs, the mean TEWL value gradually recovered to its initial level 24 h following the removal of MNs.

### 3.5. Dissolution of Needles Following Application of MNs

**Figure 5:**
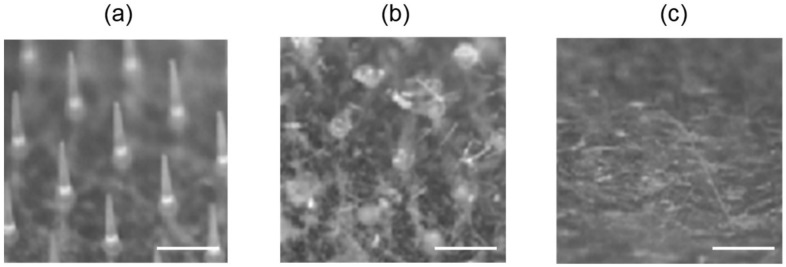
Dissolution process of needles after application of human parathyroid hormone (1-34) (PTH)-loaded microneedle arrays (MNs). (**a**) Before application, (**b**) 30 min, and (**c**) 60 min after application. Scale bars represent 800 μm.

Figure 5 shows micrograph images of needles before and after in vivo application of PTH-loaded MNs onto rat skin for 30 and 60 min. The needles, which were 800 µm in height, were clearly observed before the application. The needles were found to have mostly dissolved 30 min after the application and had completely disappeared by 60 min following application.

### 3.6. Pharmacokinetics after Application of PTH-Loaded MNs

**Figure 6:**
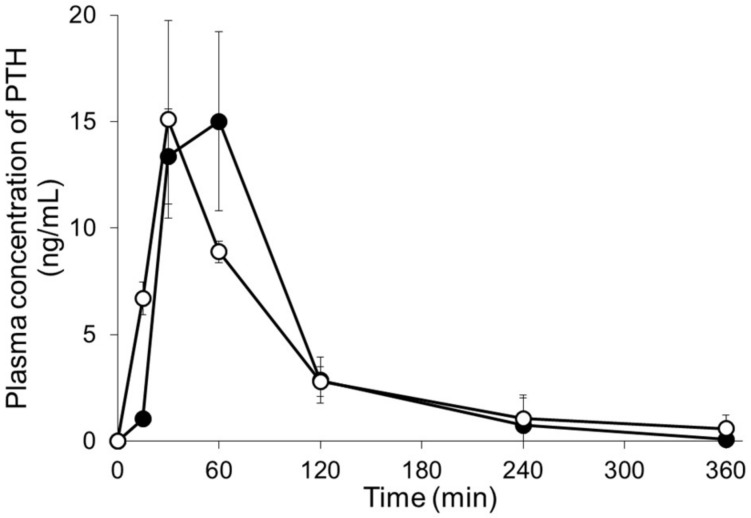
Plasma concentration-time profiles of human parathyroid hormone (1-34) (PTH) following either application of microneedle arrays (MNs) or subcutaneous injection. Keys: (●) PTH-loaded MNs and (〇) subcutaneous injection of PTH. Results are expressed as the means ± standard deviation (SD) of three rats.

Figure 6 and Table 1 show the plasma concentration profiles and pharmacokinetic parameters of PTH following either application of MNs or subcutaneous injection. Although the time to peak plasma PTH concentration (T_max_) following application of PTH-loaded MNs was slightly delayed compared with that observed after subcutaneous injection, the plasma concentrations of PTH rapidly increased following application of PTH-loaded MNs in a manner similar to that observed following subcutaneous injection. The AUC_0–∞_ values of PTH were 1354 ± 484 and 1355 ± 54 ng·mL/min after the application of MNs and after subcutaneous injection, respectively. The bioavailability (BA) of PTH relative to subcutaneous injection was 100 ± 4% after application of PTH-loaded MNs.

### 3.7. Skin Irritation Following Application of MNs

**Figure 7:**
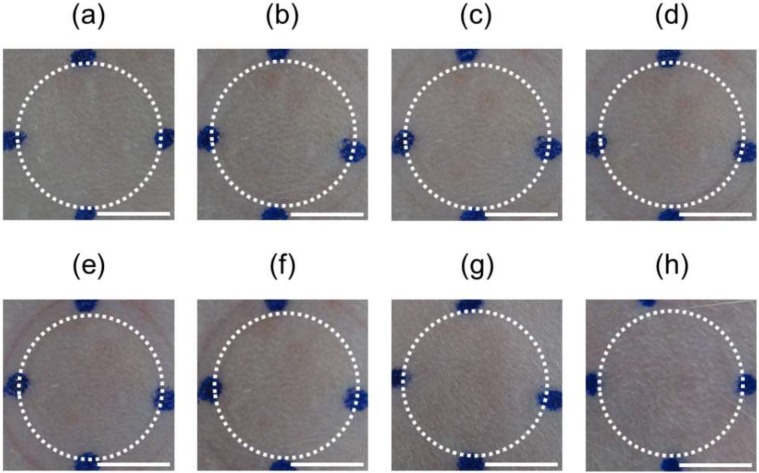
Skin irritation observed following application of human parathyroid hormone (1-34) (PTH)-loaded microneedle arrays (MNs). Typical images of rat skin (**a**) before application of PTH-loaded MNs, (**b**) immediately after removal, and (**c**) 1 h, (**d**) 2 h, (**e**) 3 h, (**f**) 4 h, (**g**) 24 h, and (**h**) 48 h after application of PTH-loaded MNs. Scale bars represent 5 mm.

Figure 7 shows the surface images of rat skin before and after application of PTH-loaded MNs. No skin erythema or edema was observed until 48 h after removal of PTH-loaded MNs.

### 3.8. Therapeutic Potential of PTH-Loaded MNs in a Rat Model of Osteoporosis

**Figure 8:**
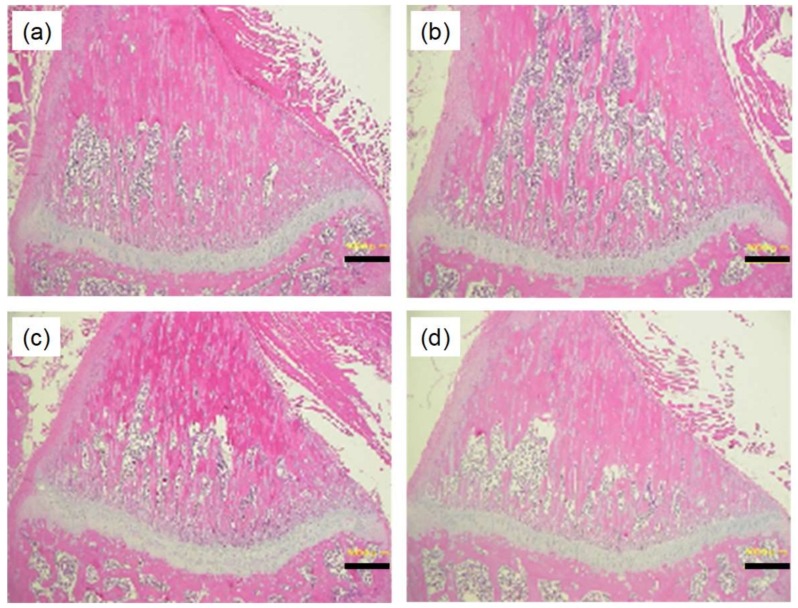
Histological micrographs of bone tissue from rat tibias following application of human parathyroid hormone (1-34) (PTH)-loaded microneedle arrays (MNs) in rats with ovariectomy (OVX)-induced osteoporosis. (**a**) Naïve, (**b**) OVX, (**c**) subcutaneous administration of PTH, and (**d**) PTH-loaded MNs. Scale bars represent 500 μm.

Figure 8 shows the effect of PTH-loaded MNs on bone structure and density in a rat model of osteoporosis. The bone matrix (shown in pink) was found to be reduced 8 weeks after OVX operation, indicating that osteoporosis was induced. The decrease in bone matrix density was effectively prevented by the subcutaneous injection of PTH in OVX rats. Similarly, PTH-loaded MNs effectively suppressed the decrease of bone matrix density after application.

## 4. Discussion

In the present study, we developed dissolving MNs fabricated from HA for the efficient transdermal delivery of PTH. HA has been widely used in the manufacturing of drug delivery and cosmetic products [27,28,29] and has several advantages with regard to biocompatibility, skin safety and antimicrobial activity [30,31,32]. Therefore, we selected HA as the base material for the dissolving MNs for the present study. To the best of our knowledge, this is the first study to date that demonstrates transdermal delivery of PTH using dissolving MNs.

To evaluate the MNs in terms of their potential clinical use, a dosage of PTH equivalent to the clinical dose (20 µg) was enclosed in the MNs in the present study. Although it has previously been reported that needle strength decreases as the amount of enclosed drug is increased [17,33], our MNs displayed sufficient needle strength to pierce the rat skin even when containing the clinical dose of PTH. The results of the storage stability study carried out at three different temperatures indicate that PTH had a higher degree of stability in the MNs than in the solution. It has previously been reported that PTH can be degraded by oxidation and humidity [23,34]. The high stability of PTH-loaded MNs is probably due to the fact that PTH-loaded MNs were dried sufficiently and packaged into aluminum sachets purged with nitrogen. In general, drugs must reach the dermis for efficient delivery into systemic circulation. The mean thickness of the stratum corneum and epidermis is approximately 10–20 and 70–100 µm, respectively [35]. In the present study, the depth of pores on the skin surface was considerably shorter than the total length of the MNs, because of skin elasticity and deformation. However, we found that the depth of pores was approximately 150 µm following application of PTH-loaded MNs, which is in good agreement with the results in our previous studies [18,21,22,36], suggesting that the MNs were able to penetrate the stratum corneum and epidermis and reach the upper dermis. These results indicate that PTH-loaded MNs create pathways to reach the dermis for the efficient transdermal delivery of PTH. TEWL is known as an index for the assessment of barrier function of the stratum corneum and is used to evaluate the disruption of stratum corneum by MNs [37,38]. In the present study, the transient increase of TEWL indicates the disruption of the stratum corneum following the application of PTH-loaded MNs and the barrier function could be recovered to the initial level with the skin turnover. These results correlate well with the observation of micron-scale pores on the skin surface after the application of PTH-loaded MNs.

Daddona et al. reported that the bioavailability of PTH relative to subcutaneous injection (FORTEO^®^) was 37.4% following application of the PTH-coated titanium microneedle patch system (ZP-PTH^®^) [3,23]. This low BA was likely due to the short needle length used (190 µm). According to previous reports, the needles were partly penetrated into the skin after application of MNs, as a result of skin deformation [21,39]. Therefore, we think that the short needles in ZP-PTH^®^ would not be able to reach the upper dermis where blood vessels are present. It was reported that MNs with longer needles (>600 µm length) showed effective transdermal drug delivery [40,41]. Therefore, in the present study, MNs with 800 µm in length were used in order to achieve an efficient transdermal delivery of PTH. In the present study, the efficient transdermal delivery of PTH (BA; 100 ± 4%) was successfully achieved by MNs with 800 µm in length, although the T_max_ after application of MNs was slightly delayed compared with that after subcutaneous injection of PTH. This was probably caused by the dissolution process of MNs in the skin following application [20].

It has previously been reported that PTH has pharmacological activities for both bone formation and resorption. A previous study reported that bone formation was observed following intermittent treatment of rats with PTH [1,42]. In the present study, bone formation was observed by application of PTH-loaded MNs twice a week, and the therapeutic effects of PTH-loaded MNs on osteoporosis were proportional to the pharmacokinetics of PTH after application of PTH-loaded MNs. These results indicate that pharmacologically active PTH was effectively delivered to the bone through the skin and that application of PTH-loaded MNs enhanced bone formation.

We also demonstrated that PTH-loaded MNs were relatively safe for application to the rat skin. The results of the skin irritation study, together with the results of TEWL, indicate that PTH-loaded MNs transiently created a pathway for the transdermal delivery of PTH and caused minimal skin irritation. HA is known to be both an endogenous and highly biocompatible compound and hardly caused irritation to the rat skin. Although further studies are needed to evaluate the safety of PTH-loaded MNs in detail for potential clinical use, PTH-loaded MNs show promise as formulations for inclusion in a safe and efficient transdermal delivery system.

## 5. Conclusions

In conclusion, we developed dissolving MNs fabricated from HA as the base material for the transdermal delivery of PTH. The storage stability of PTH in MNs was much higher than that of PTH solution kept at room temperature (15–20 °C) for one month. Efficient transdermal delivery of PTH and treatment of osteoporosis were successfully achieved by application of PTH-loaded MNs without the development of any skin irritation. These findings indicate that our dissolving MNs are promising formulations for the transdermal delivery of PTH and the treatment of osteoporosis.

## Figures and Tables

**Table 1:** Pharmacokinetic parameters of human parathyroid hormone (PTH) after administration of PTH-loaded microneedle arrays (MNs) or subcutaneous injection of PTH.

	Dose (µg/rat)	*C*_max_ (ng/mL)	*T*_max_ (min)	AUC_0–∞ min_ (ng·mL/min)	BA (%)
PTH s.c.	20	15.1 ± 4.6	30 ± 0	1354 ± 484	—
PTH-loaded MNs	20	15.3 ± 3.6	50 ± 17	1355 ± 54	100 ± 4

s.c., subcutaneous injection; AUC, area under the plasma concentration-time curve; BA, bioavailability.
